# Clinical acceptability of fully automated external beam radiotherapy for cervical cancer with three different beam delivery techniques

**DOI:** 10.1002/mp.15868

**Published:** 2022-07-26

**Authors:** Dong Joo Rhee, Anuja Jhingran, Kai Huang, Tucker J. Netherton, Nazia Fakie, Ingrid White, Alicia Sherriff, Carlos E. Cardenas, Lifei Zhang, Surendra Prajapati, Stephen F. Kry, Beth M. Beadle, William Shaw, Frederika O'Reilly, Jeannette Parkes, Hester Burger, Chris Trauernicht, Hannah Simonds, Laurence E. Court

**Affiliations:** ^1^ Medical Physics The University of Texas Graduate School of Biomedical Sciences at Houston Houston Texas USA; ^2^ Department of Radiation Physics, Division of Radiation Oncology The University of Texas MD Anderson Cancer Center Houston Texas USA; ^3^ Department of Radiation Oncology The University of Texas MD Anderson Cancer Center Houston Texas USA; ^4^ Division of Radiation Oncology and Medical Physics University of Cape Town and Groote Schuur Hospital Cape Town South Africa; ^5^ Radiotherapy Department Guy's and St Thomas’ NHS Foundation Trust London UK; ^6^ Department of Oncology University of the Free State Bloemfontein South Africa; ^7^ Department of Radiation Oncology The University of Alabama at Birmingham Birmingham Alabama USA; ^8^ Department of Radiation Oncology Stanford University School of Medicine Stanford California USA; ^9^ Department of Medical Physics (G68) University of the Free State Bloemfontein South Africa; ^10^ Division of Medical Physics Tygerberg Academic Hospital Stellenbosch University Cape Town South Africa; ^11^ Division of Radiation Oncology Tygerberg Academic Hospital Stellenbosch University Cape Town South Africa

**Keywords:** auto‐contouring, auto‐planning, cervical cancer, field‐in‐field

## Abstract

**Purpose:**

To fully automate CT‐based cervical cancer radiotherapy by automating contouring and planning for three different treatment techniques.

**Methods:**

We automated three different radiotherapy planning techniques for locally advanced cervical cancer: 2D 4‐field‐box (4‐field‐box), 3D conformal radiotherapy (3D‐CRT), and volumetric modulated arc therapy (VMAT). These auto‐planning algorithms were combined with a previously developed auto‐contouring system. To improve the quality of the 4‐field‐box and 3D‐CRT plans, we used an in‐house, field‐in‐field (FIF) automation program. Thirty‐five plans were generated for each technique on CT scans from multiple institutions and evaluated by five experienced radiation oncologists from three different countries. Every plan was reviewed by two of the five radiation oncologists and scored using a 5‐point Likert scale.

**Results:**

Overall, 87%, 99%, and 94% of the automatically generated plans were found to be clinically acceptable without modification for the 4‐field‐box, 3D‐CRT, and VMAT plans, respectively. Some customizations of the FIF configuration were necessary on the basis of radiation oncologist preference. Additionally, in some cases, it was necessary to renormalize the plan after it was generated to satisfy radiation oncologist preference.

**Conclusion:**

Approximately, 90% of the automatically generated plans were clinically acceptable for all three planning techniques. This fully automated planning system has been implemented into the radiation planning assistant for further testing in resource‐constrained radiotherapy departments in low‐ and middle‐income countries.

## 1 INTRODUCTION

Radiotherapy is a curative treatment for cancer, with different delivery techniques available for use. Radiation oncologists choose the optimal technique considering the patients’ condition, the capabilities of the radiation equipment they possess, their familiarity and confidence with the technique, and factors such as patient‐throughput and the resources required to perform patient‐specific quality assurance. For cervical cancer, the most common beam delivery techniques for external‐beam radiotherapy include 2D 4‐field box (4‐field‐box), 3D conformal radiotherapy (3D‐CRT), intensity‐modulated radiotherapy (IMRT), and volumetric modulated arc therapy (VMAT). Several studies have been conducted to automate such techniques and validate the performance of the automation tools to improve the efficiency of the planning process and the plan quality.

The automation algorithm of the 4‐field‐box technique for cervical cancer was developed by Kisling et al.^1^ The beam apertures were determined using the bony landmarks in 2D‐projected CT scans for each gantry angle, and the bony structures were automatically contoured using a multi‐atlas–based auto‐contouring system. IMRT or VMAT plans can be automatically generated using the commercially available knowledge‐based planning software programs, such as RapidPlan (Varian Medical Systems, Palo Alto, CA) and Erasmus‐iCycle (Elekta AB, Stockholm, Sweden). The performance of knowledge‐based planning models for cervical cancer has been validated in multiple studies. Ma et al.^2^ tested an IMRT RapidPlan model in patients with cervical cancer treated with surgery; the planning target volume (PTV) coverage was within 1%, and critical organ dose metrics were within 4% of the manual plan results. Li et al.^3^ and Tinoco et al.^4^ showed that the IMRT and VMAT RapidPlan models for patients with cervical cancer were superior or equivalent to clinical plans. Sharfo et al.^5^ showed that their dual‐arc VMAT Erasmus‐iCycle model created plans were superior or equivalent to manually generated dual‐arc VMAT and 9‐beam IMRT for patients with cervical cancer. Thus, automatically generated IMRT or VMAT plan for cervical cancer developed using knowledge‐based planning techniques have demonstrated clinical acceptability when high‐quality plans are used for model training. However, most of these studies were not fully automated, as they used manually generated contours to create the plans.

In this study, we developed end‐to‐end solutions that can automatically generate 4‐field‐box, 3D‐CRT, and VMAT plans for cervical cancer. We also used the in‐house field‐in‐field (FIF) automation algorithm^6^ to improve the quality of the 4‐field‐box and 3D‐CRT plans. Unlike most of the auto‐planning studies described above, in which the plans are generated using manual contours, we combined the auto‐planning algorithms with the auto‐contouring system described in our previous study^7^ to fully automate the radiotherapy plan generation process for cervical cancer with minimal human input. The quality of the plans was evaluated by multiple radiation oncologists from various countries. Our fully automated radiotherapy planning system for cervical cancer has been implemented in the radiation planning assistant (RPA)^8^ system to further testing in resource‐constrained radiation oncology departments in low‐ and middle‐income countries. The RPA system would be able to accelerate the radiotherapy planning process in these clinics, and therefore more patients in low‐ and middle‐income countries can benefit from it.

## 2 METHODS

We developed auto‐planning systems for cervical cancer using three different treatment techniques: 4‐field‐box, 3D‐CRT, and VMAT. The auto‐planning systems were developed to treat cervical cancer patients with an intact uterus and no vaginal or para‐aortic nodal (PAN) involvement. The systems were integrated with the auto‐contouring system^7^ to fully automate radiotherapy planning for cervical cancer on CT images. The only human inputs used to generate the plans were: upload and verification of the CT images, prescription of dose, and determination of the margins for the internal tumor volume (ITV), the PTV, and the beam aperture. For the beam energy, we only used 6 MV photon beams to create the plans, as higher photon beam energies might not be available in certain clinics in low‐ and middle‐income countries. The anisotropic analytical algorithm (AAA) implemented in the Eclipse (Varian Medical Systems, Palo Alto, CA) treatment planning system was used for dose calculation for all three techniques.

### 2.1 4‐Field‐box plans from bony landmarks

The beam apertures for the 4‐field‐box plans were determined on the basis of the algorithm from Kisling et al.^1^, ^9^ In this study, we replaced the multi‐atlas‐based auto‐contouring system from the previous study with our deep‐learning‐based auto‐contouring system,^7^ which has the better performance in our preliminary study. The 3D contours of the pelvic bones, the sacrum, the left and right femurs, and the L4 and L5 lumbar vertebral bodies were generated on CT images. The contours were projected to 0°, 90°, 180°, and 270° gantry angles. In each projection angle, certain bony landmarks from the projected contours were detected, and the beam apertures were shaped on the basis of these bony landmarks.

**FIGURE 1:**
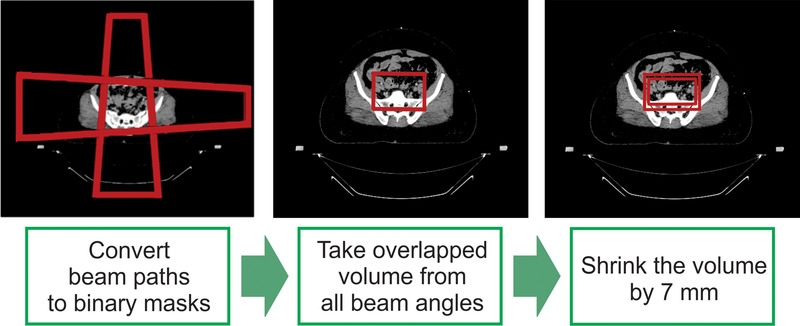
The synthetic planning target volume (PTV) structure was defined on the basis of the beam apertures for the 4‐field‐box plans. First, the geometric beam path from each beam angle was converted into a 3D binary mask, and then the volume overlapped by each mask was defined as the region of hot spot detection (RHD). Finally, the synthetic PTV was created from 7 mm shrinkage of the RHD

In clinical practice, a calculation point is often used to normalize the plan. This point is arbitrarily determined by the clinician on the basis of patient geometry and initial dose distribution. As it is challenging to automate the determination of the calculation point, we defined a volume called “synthetic PTV” and normalized the plan using this volume instead. The synthetic PTV was defined as the volume overlapped by the beam paths and then shrunk by 7 mm uniformly, as shown in Figure 1. We subtracted 7 mm from the overlapped volume because the beam shape was determined by the projected PTV plus a 7 mm uniform margin for 3D‐CRT plans. All of the plans were normalized such that 100% of the prescription dose covers 97% of the synthetic PTV initially, as this gives similar dose distribution with the plans when 100% of the prescription dose covering 95% of the actual PTV if the PTV contour is available.

### 2.2 3D‐CRT plans with CTV contours

**FIGURE 2:**
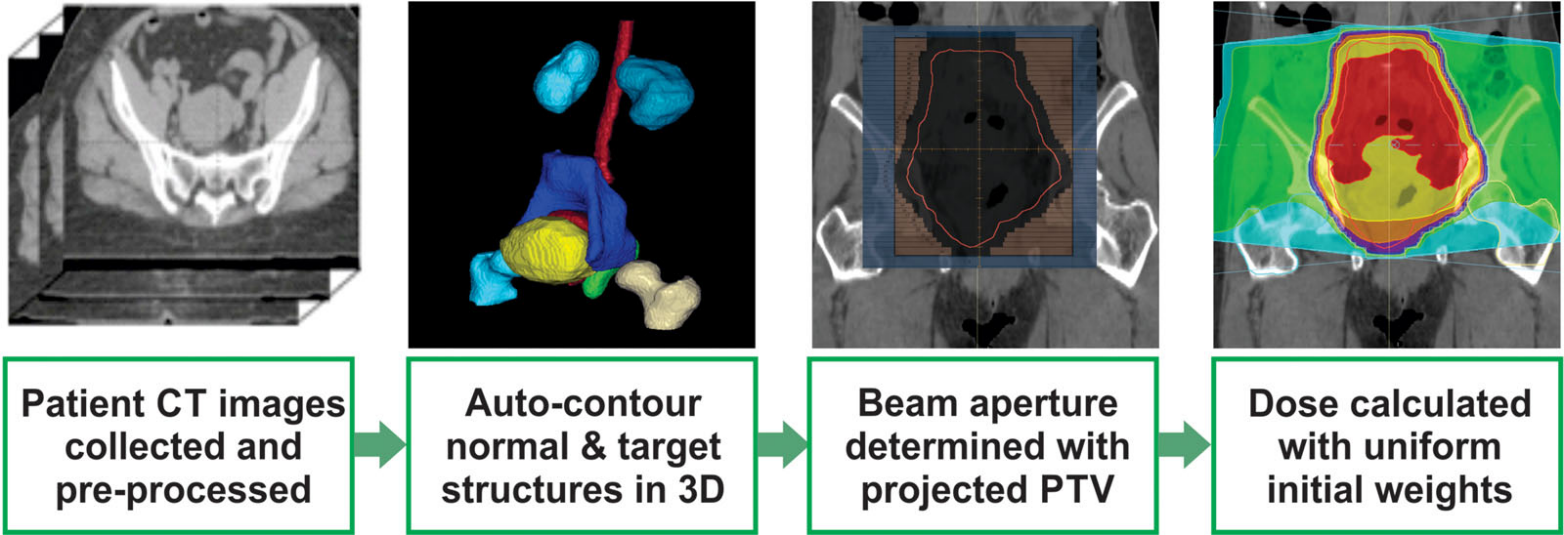
Workflow of the automated 3D conformal radiotherapy (3D‐CRT) system for cervical cancer. The planning target volume (PTV) was derived from the automatically generated clinical target volumes. The beam apertures were determined with a user‐defined uniform margin (7 mm in this study) around the projected PTV. The dose was calculated with a pre‐defined MU

The beam apertures for the 3D‐CRT plans were determined using the projected PTV contours from 0°, 90°, 180°, and 270° gantry angles. The primary and nodal clinical target volumes (CTV) were generated using the auto‐contouring system, and the PTV was an expansion of the CTVs with the basic image‐guided radiotherapy margins for the primary CTV, as described in the GEC‐ESTRO EMBRACE II protocol (10 mm in the anterior, posterior, superior, and inferior directions and 5 mm in the lateral directions),^10^, ^11^ and an additional 5 mm PTV margin. Finally, a 7 mm uniform margin was applied to the projected PTV to determine the beam shape at each gantry angle. The step‐by‐step process of generating the 3D‐CRT plan is demonstrated in Figure 2. The plans were normalized such that 100% of the prescription dose covers 95% of the PTV initially.

Even with correctly generated beam apertures for the 4‐field‐box or 3D‐CRT plans, the plans might not be clinically acceptable because of hot spots. In this study, hot spots were defined as any volume larger than 2 cc that received more than 107% of the prescription dose. To reduce the hot spots and make the plans more clinically acceptable, we used the in‐house FIF automation program.^6^ Dose–volume constraints can be easily modified by changing the parameters in the configuration file of the FIF automation program. The minimum MU for each FIF segment was set to be 7 MU.

### 2.3 VMAT

We trained a commercially available solution (Varian RapidPlan) to automatically generate VMAT plans for cervical cancer. To train the RapidPlan model, we generated VMAT plans on 97 retrospective cervical cancer patients. The RapidPlan model can treat up to three dose levels and uses 6‐MV photon beams, three full arcs, and three collimator angles, including 10°, 90°, and 350°. For planning objectives, we used automatically generated bladder, bowel space, femurs, kidney, liver, rectum, spinal cord, and bone marrow contours; the bone marrow contour was defined as the summation of the pelvis, sacrum, femurs, and L5 vertebral body. The planning objectives of the PTV were set to achieve 95% of the PTV covered by 100% of the prescription dose, while the maximum dose was <107% of the prescription dose.

### 2.4 Plan review

Radiotherapy plans were generated on 35 CT scans from three different South African hospitals using each of the three planning techniques. In total, five experienced radiation oncologists (three radiation oncologists from South Africa, one from the United States, and one from the United Kingdom) scored these plans, and each plan for each technique was scored by two of these radiation oncologists. The plans were assigned to radiation oncologists such that they only reviewed the techniques that they typically used in clinical practice. We used the Likert scale, a 5‐scale scoring system where 1 being the unusable plan and 5 being the perfect plan, to evaluate the plans, as defined in Table 1.

**TABLE 1:** Likert scale to score automatically generated radiotherapy plans

**Score**		**Description**
5	Strongly agree	Use as‐is. Clinically acceptable. Plans can be used for treatment without change.
4	Agree	Minor edits that are not necessary. Stylistic changes preferred, but not clinically important. Current plans are clinically acceptable.
3	Neither agree nor disagree	Minor edits that are necessary. Minor edits are those that can be made in less time than starting from scratch or are expected to have minimal effect on treatment outcome.
2	Disagree	Major edits. Necessary edits are required to ensure appropriate treatment and are sufficiently significant that the user would prefer to start from scratch.
1	Strongly disagree	Unusable. Quality of the automatically generated plans is so bad that they are unusable.

We showed a small initial set of plans to each radiation oncologist first to determine whether they were satisfied with the plan quality. If the plans were consistently scored low for the same reasons (e.g., being too hot or too cold), we adjusted all plans by changing the FIF parameters or by renormalization on the basis of the radiation oncologist's preference. For example, for the 3D‐CRT plans, one of the reviewers found the plans to be too hot and wanted 95% of the PTV to be covered by 95% of the prescription dose instead of 100% of the prescription dose. These variations were mostly originated from different practices in each clinic (e.g., prescription dose being either 45 Gy or 50 Gy). After adjusting the plans on the basis of the radiation oncologists’ preferences, we asked them to review the plans from the beginning.

## 3 RESULTS

The overall review results are shown in Table 2. Thirty‐five plans were created for each treatment technique. The reviewer number in the table was randomly assigned to indicate that two radiation oncologists reviewed each set of plans independently. The average time (±1σ) to create a plan was 9.2 ± 2.3 min, 8.5 ± 2.6 min, and 46.6 ± 8.6 min for 4‐field‐box, 3D‐CRT, and VMAT, respectively.

**TABLE 2:** Radiation oncologist scoring results for each technique with each review session. The plan criteria for the coverage and the maximum dose are presented. The reviewer numbers were arbitrarily assigned

**Treatment technique**	**Reviewer #**	**Coverage (Rx/PTV)**	**Max dose (%)**	**# of plans in each score**				
				**5 (Best)**	**4**	**3**	**2**	**1 (Worst)**
4‐Field‐box	1	100%/97%	107	9	20	5	0	1
	2	100%/97%	105	28	4	2	0	1
3D‐CRT	1	100%/95%	107	27	7	1	0	0
	2	95%/99%	105	3	32	0	0	0
VMAT	1	100%/95%	107	16	15	3	1	0
	2	95%/99%	107	35	0	0	0	0

### 3.1 4‐Field‐box

Reviewer #1 was satisfied with the original plans, where the maximum dose was <107% of the prescription dose. This reviewer scored 29 out of the 35 plans as clinically acceptable without modification (score ≥4), and five plans as minor edits required due to excessive bowel dose. One plan was scored as 1 as a result of a failure to generate correct L4 contours; therefore, the superior borders of the beam apertures were incorrectly defined.

**FIGURE 3:**
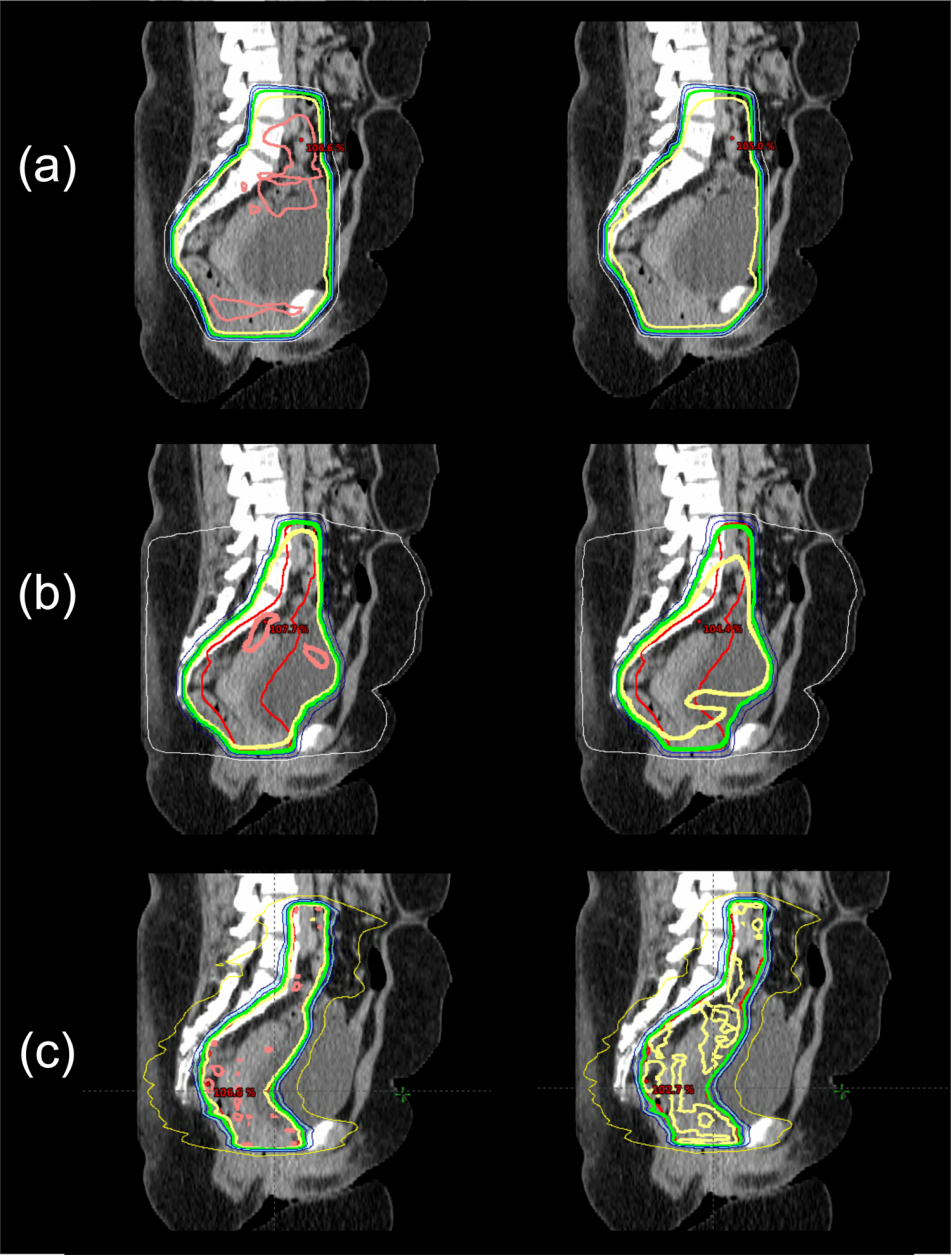
Demonstration of the customized plans for Reviewer #1 (left) and Reviewer #2 (right) for the (a) 4‐field‐box, (b) 3D conformal radiotherapy, and (c) volumetric modulated arc therapy techniques in three patients with intermediate risk cervical cancer. The thick lines represent the planning target volume (red), 105% isodose line (pink), 100% isodose line (yellow), and 95% isodose line (green)

Reviewer #2 requested the dose >105% to be minimized and not be in the rectum, the hotspot constraint in the FIF program was altered from 107% to 105%. Consequently, most of the 105% isodose lines from the original plan were removed from the updated plan, as shown in Figure 3a, although the number of sub‐fields from the FIF program was increased. This reviewer scored 32 plans as clinically acceptable without modification. Two plans were scored as 3 because of insufficient PTV coverage. The plan that was scored as 1 was the same plan that was identified by the first reviewer.

### 3.2 3D‐CRT

Reviewer #1 was satisfied with the original plan, where 95% of the PTV was covered by 100% of the prescription dose. This reviewer scored 34 plans as clinically acceptable without modification. One plan was scored as 3 because portions of the PTV were not covered by the prescription dose.

Reviewer #2 found the original plans to be too hot and preferred to have almost all (99%) of the PTV covered by 95% of the prescription dose. Furthermore, this reviewer wanted to remove all areas of 70% dose outside of the main treatment region (i.e., RHD). We adjusted all of the plans by changing the parameters in the FIF program on the basis of these criteria, and the updated plans were cooler than the original plans, as shown in Figure 3b. After the adjustment, this reviewer scored all 35 plans as clinically acceptable without modification (score ≥4). The reviewer scored most of the plans as 4, mostly because the PTV in the most inferior slice was not fully covered by 95% of the prescription dose.

For quantitative evaluation, we analyzed V40Gy for the bladder, rectum, femurs, and bowel space, and the maximum dose to the spinal cord for the 3D‐CRT plans optimized to cover 95% of the PTV with 100% prescription dose. The average V40Gy was 88.9 ± 8.9% for the bladder, 94.8 ± 5.4% for the rectum, 2.5 ± 1.8% for the femurs, and 27.2 ± 6.6% for the bowel space. The average maximum dose to the spinal cord was 37.2 ± 5.7 Gy.

### 3.3 VMAT

Reviewer #1 was satisfied with the original plan in which the planning objectives were set to have 95% of the PTV covered by 100% of the prescription dose. This reviewer checked the overall dose distribution instead of relying on certain dose metrics for the organs at risk (OARs). The reviewer scored 31 plans as clinically acceptable without modification (score ≥4). Four plans were scored as 2 and 3 because of insufficient PTV coverage.

Reviewer #2 preferred the PTV to be covered by 95% of the prescription dose. We renormalized the plans such that 99% of the PTV was covered by 95% of the prescription dose to meet Reviewer #2′s preferences. This renormalization usually made the plans cooler than the original plans, as shown in Figure 3c. The dose metrics for the OARs, including the bladder, bowel, femurs, and rectum, were also considered, although some of the metrics for the bowel and rectum were accepted if greater than the preset dose constraints when the PTV was substantially overlapped with these structures. This reviewer scored all 35 plans as 5.

**FIGURE 4:**
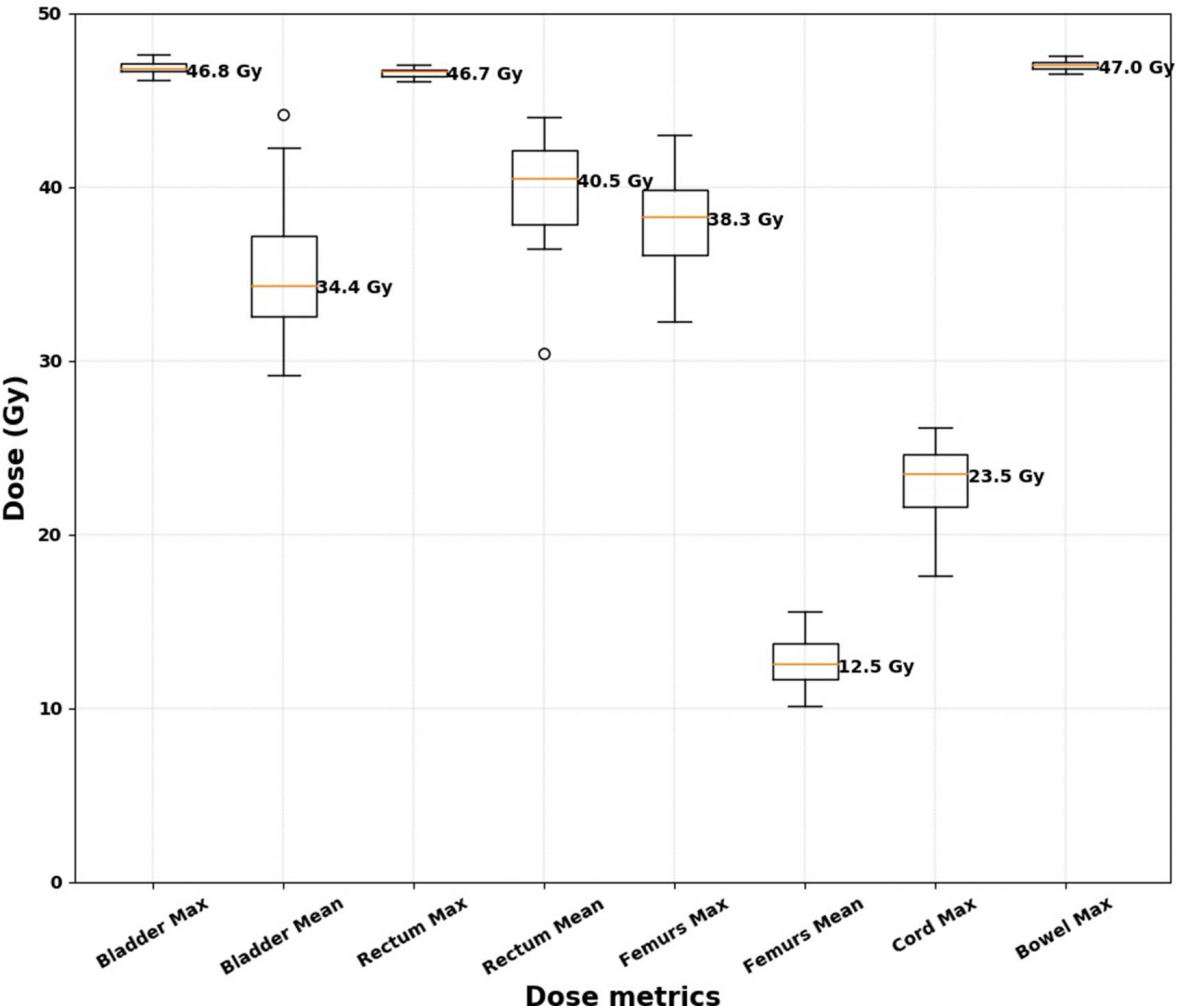
Boxplots for the normal structure dose metrics for the 35 volumetric modulated arc therapy plans. The median values for the dose metrics are indicated next to each boxplot

**FIGURE 5:**
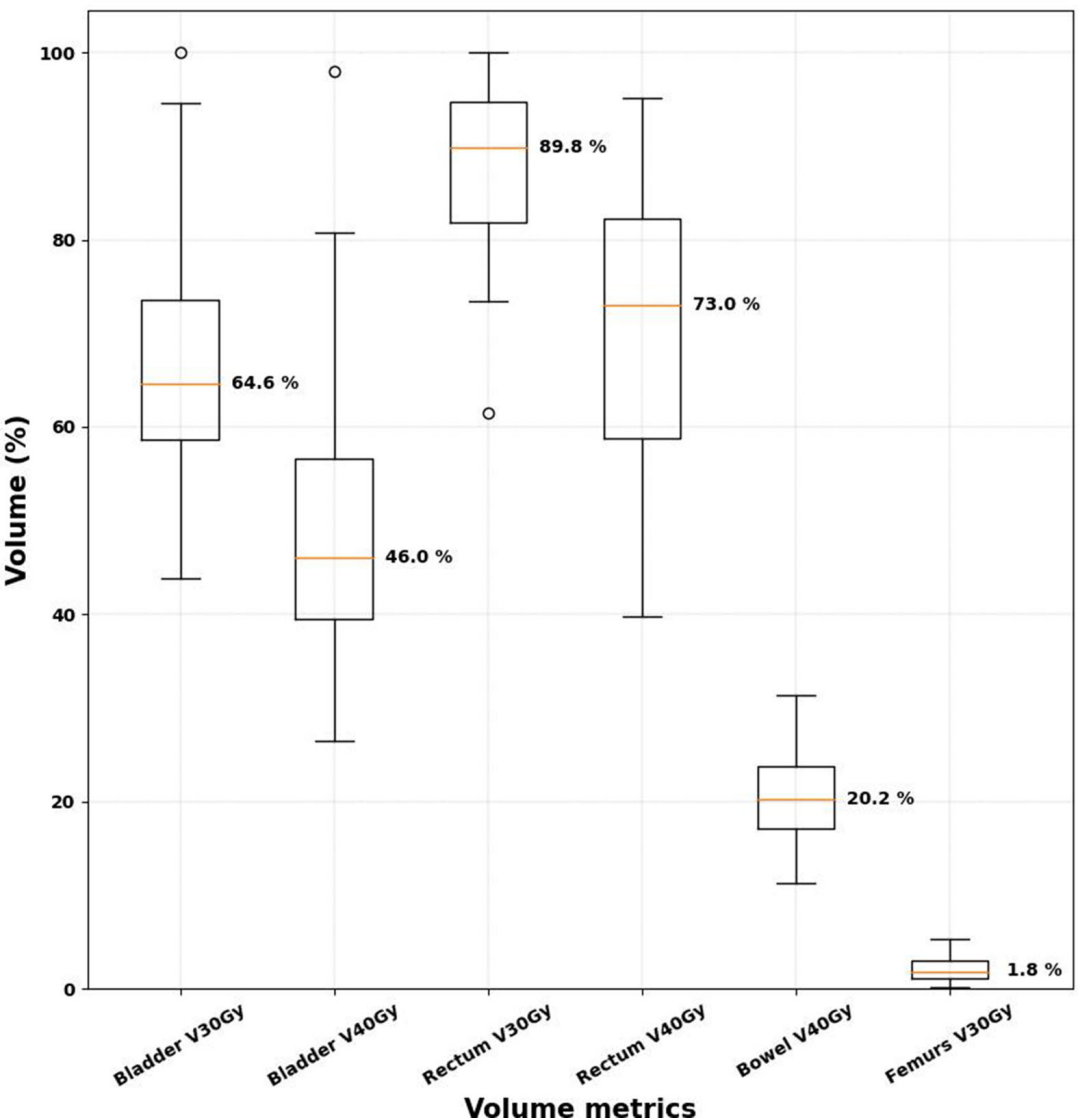
Boxplots for the normal structure dose–volume metrics for the 35 volumetric modulated arc therapy plans. The median values for the volume metrics are indicated next to each boxplot

We calculated the average and maximum doses of the bladder, rectum, and femurs, and the maximum dose of the spinal cord and bowel space for VMAT plans, as shown in the boxplots in Figure 4. The calculated values are all lower than the hard dose constraints in the GEC‐ESTRO EMBRACE II protocol, where the maximum dose constraints for the bladder, rectum, femurs, spinal cord, and bowel are all less than 105% of the prescription dose (47.3 Gy). Furthermore, we calculated the common soft dose constraints for the OARs, including V30Gy and V40Gy for the bladder and rectum, V40Gy for the bowel space, and V30Gy for the femurs, as shown in Figure 5. In the GEC‐ESTRO EMBRACE II protocol, V40Gy < 75% and 85%, and V30Gy < 85% and 95% for the bladder and rectum, respectively, and in our internal protocol, V45Gy < 50% and 80% for the bladder and rectum, respectively. Therefore, the soft tissue constraints for the majority of the automatically generated plans were met for the bladder and rectum. V40Gy for the bowel space is less than 30% and V40Gy for the femurs is less than 15% in our internal soft dose constraints, and these metrics from all of the auto plans were lower than the soft dose constraints. The soft dose constraints for the bowel in the EMBRACE II protocol (V40Gy < 100cc and V30Gy < 350cc) were not assessed as the definition of the bowel contour from the protocol was the actual bowel loops, not the entire bowel space like our definition of the bowel.

## 4 DISCUSSION

Overall, 87%, 99%, and 94% of the automatically generated plans were found to be clinically acceptable without modification (score ≥4) for the 4‐field‐box, 3D‐CRT, and VMAT plans, respectively. Although adjustment was made to meet each radiation oncologist's preference, this adjustment can be easily achieved by renormalizing the plan in the treatment planning system or changing the parameters in the configuration files of the FIF automation program. For the plans scored ≤3, we obtained feedback from the reviewers and carefully revisited the concerns. The overall performance of the auto‐contouring system was independently evaluated in our previous study.^7^

### 4.1 4‐Field‐box

**FIGURE 6:**
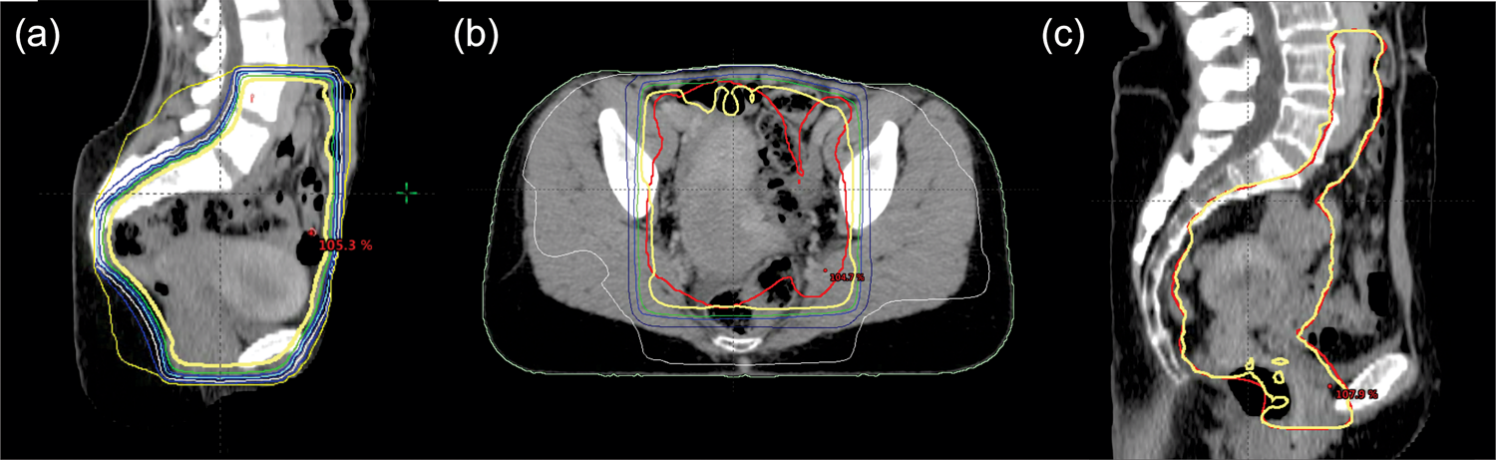
Examples of the plans scored ≤3. The thick yellow lines represent the 100% isodose line, and the thick red lines represent the planning target volume (PTV). (a) The 4‐field‐box plan was scored as 3 because of the excessive dose to the bowel. (b) The 3D conformal radiotherapy plan was scored as 3 as the PTV (red) was not fully covered by a 100% isodose line near the bowel. (c) The volumetric modulated arc therapy plan was scored as 2 as the PTV (red) was not fully covered by a 100% isodose line near the rectum

Five 4‐field‐box plans were scored as 3 by Reviewer #1 because of the excessive dose to the bowel. Four of these five patients had low body mass index (BMI), as shown in Figure 6a. The remaining patient had normal BMI, but the patient was wide in the lateral direction and narrow in the anterior–posterior direction. These patients have very limited space between the target and the bowel. The beam apertures for the 4‐field‐box plans were determined on the basis of the bony landmarks, not the soft tissue structures. These bony landmarks were chosen to not miss the targets in most patients; therefore, the method used to determine the beam shapes was not optimized to spare the dose to the bowel. As a result, the beam apertures of the 4‐field‐box plans are likely to excessively cover the bowel region in low BMI patients. This is a limitation of the 4‐field‐box approach, not the automation system; therefore, we did not count these cases in our performance evaluation.

In one case, the 4‐field‐box plan was scored as 1 as a result of the incorrectly generated L4 contour. In our preliminary study, we asked radiation oncologists to score the automatically generated beam apertures of 103 patients. Three of these apertures were evaluated as clinically unacceptable; all of the failures were due to the incorrectly created L4 and/or L5 contours. Therefore, the overall acceptance rate for the automatically generated 4‐field‐box plans was around 97% from these two studies. Many of the failures in the L4/L5 contours were due to atypical vertebral counts (sacralization or lumbarization), which are very challenging for the deep‐learning‐based auto‐contouring systems to detect.^12^ This was the limitation of the 4‐field‐box auto‐planning system. For these cases, clinicians are required to manually modify the incorrectly generated contours, or they can use automatically generated 3D‐CRT plans instead.

### 4.2 3D‐CRT

Almost all of the 3D‐CRT plans were scored ≥ 4. Reviewer #2 scored one plan as 3 as the PTV was not covered well and the 100% isodose line was not smooth, as shown in Figure 6b. For this patient, achieving good PTV coverage and a smooth isodose line was challenging, as some parts of the PTV were located too close to the surface of the body and the bowel was filled with gas.

Reviewer #1 scored most plans as 4 as the most inferior slices were not fully covered by the 95% isodose line. Similarly, some plans were scored as 4 by Reviewer #2, mainly because the most inferior slices were not fully covered by the 100% isodose line. As the 3D‐CRT plans were naively normalized by the PTV, the dose near the edges of the beam apertures, especially the most superior and inferior slices, was often colder than the rest of the PTV. In a future work, we will further improve the in‐house FIF automation program so that the PTV in the most inferior slice can be fully covered by the desired isodose line.

### 4.3 VMAT

For the VMAT plans, Reviewer #1 scored four of the plans as either 2 or 3. These plans did not have good PTV coverage, as shown in Figure 6c. Three did not have good PTV coverage near the rectal region because of the gas‐filled rectum, as shown in Figure 6c. The CT scans with gas‐filled rectums with a diameter larger than 4 cm are not ideal for radiotherapy planning, according to the GEC‐ESTRO EMBRACE II protocol^10^, ^11^; therefore, we believe that the clinical acceptance rate can vary depending on how strictly the simulation protocol is followed.

### 4.4 OAR dose metrics and safety

To ensure that the doses to the OARs from our plans were similar to those from the manually generated 3D‐CRT and VMAT plans, we compared V40Gy of the bladder, rectum, and femurs from other studies. For the bladder, V40Gy of our auto‐plans were 88.9 ± 8.9% and 50.3 ± 15.8% for 3D‐CRT and VMAT, respectively. These are comparable to the results from Deng et al.^13^ (92.8 ± 10.1% and 37.3 ± 6.3%), or Guy et al.^14^ (88.4 ± 13.0% and 58.6 ± 24.0%). For the rectum, V40Gy of our auto‐plans were 94.8 ± 5.4% and 70.7 ± 14.1% for 3D‐CRT and VMAT, respectively, and again, these are also comparable to the previous studies from Deng et al.^13^ (96.1 ± 2.9% and 44.5 ± 4.8%), Guy et al.^14^ (85.0 ± 20.3% and 72.0 ± 31.5%), or Bhagaloo et al.^15^ (88.5% and 96.0%). Similarly for the femurs, V40Gy of our auto‐plans were 2.5 ± 1.8% and 0.0 ± 0.1% for 3D‐CRT and VMAT, respectively, and those for Deng et al.^13^ (0.5 ± 1.2% and 12.7 ± 8.0%) were similar to our results.

Although the entire planning process was streamlined and fully automated in this study, it is still very important that users carefully review and edit automatically generated contours and plans before final approval.^16^ Furthermore, we are developing a range of automated quality assurance systems for contours^17^ and plans,^18^, ^19^ with the goal of identifying potential planning errors. Although simple to use, it is also important to emphasize the need for user training so that users understand the potential risks and can use the system safely. The users of the RPA system must take video‐based training if they request to have access to the system. Through this training, they understand every feature in the system as well as the limitations of the system and learned how to use the RPA system safely. Lastly, to reduce the risk of incorrect use of the customization features in the RPA auto planning system, we set the range of acceptable values for each variable. For example, the acceptable range of the prescription dose is from 43.2 Gy to 50.4 Gy, and the range of the PTV margin is from 0.3 cm to 1.0 cm. This way, the users will be free of making mistakes from typo or misreading of the units.

## 5 CONCLUSIONS

We demonstrated that the auto‐planning system, combined with the auto‐contouring system, can generate clinically acceptable plans with three different beam delivery techniques for cervical cancer radiotherapy. The plans should be optimized to meet each user's preference. Approximately 90% of the automatically generated plans were clinically acceptable for all three techniques. Furthermore, we demonstrated that the system can be tailored to meet the preferences of multiple users in different clinics. This auto‐planning system has been incorporated into the RPA to aid under‐resourced hospitals and patients in low‐ and middle‐income countries.
